# Protective Actions of 17*β*-Estradiol and Progesterone on Oxidative Neuronal Injury Induced by Organometallic Compounds

**DOI:** 10.1155/2015/343706

**Published:** 2015-03-01

**Authors:** Yasuhiro Ishihara, Takuya Takemoto, Atsuhiko Ishida, Takeshi Yamazaki

**Affiliations:** Laboratory of Molecular Brain Science, Graduate School of Integrated Arts and Sciences, Hiroshima University, Higashihiroshima 739-8521, Japan

## Abstract

Steroid hormones synthesized in and secreted from peripheral endocrine glands pass through the blood-brain barrier and play a role in the central nervous system. In addition, the brain possesses an inherent endocrine system and synthesizes steroid hormones known as neurosteroids. Increasing evidence shows that neuroactive steroids protect the central nervous system from various harmful stimuli. Reports show that the neuroprotective actions of steroid hormones attenuate oxidative stress. In this review, we summarize the antioxidative effects of neuroactive steroids, especially 17*β*-estradiol and progesterone, on neuronal injury in the central nervous system under various pathological conditions, and then describe our recent findings concerning the neuroprotective actions of 17*β*-estradiol and progesterone on oxidative neuronal injury induced by organometallic compounds, tributyltin, and methylmercury.

## 1. Introduction

Reactive oxygen species (ROS) are the general term for reactive molecules derived from oxygen, including superoxide, hydrogen peroxide, and hydroxyl radicals. ROS are generated constitutively from cellular organelles, especially mitochondria, and a variety of xenobiotics such as quinones induce ROS production. Cells have defense systems to cope with routinely generated ROS. Superoxide is dismutated to hydrogen peroxide and molecular oxygen by superoxide dismutase (SOD). SOD1 (Cu/Zn-SOD) is present in the cytoplasm, nucleus, mitochondria, and peroxisomes of all mammalian cells, where it scavenges superoxide [1, 2]. SOD2 (mitochondrial Mn-SOD) efficiently eliminates the superoxide that arises from molecular oxygen in the respiratory chain. Hydrogen peroxide produced by the dismutation of superoxide is degraded to molecular oxygen and water by catalase, glutathione peroxidase (GPx), and peroxiredoxin. Catalases are present ubiquitously in aerobic organisms, and the highest level of catalase activity is found in the liver and erythrocytes. Within cells, catalases are located mostly in the peroxisomes because of the presence of many hydrogen peroxide-producing enzymes. There are 4 types of GPx: classical GPx (GPx1), gastrointestinal GPx (GPx2), plasma GPx (GPx3), and phospholipid hydroperoxide GPx (GPx4) [3]. GPx1, GPx2, and GPx3 reduce hydrogen peroxide and organic alkyl hydroperoxides to water and corresponding alcohols at the expense of oxidation of GSH to GSSG. The last type of peroxidase, GPx4, is different from the other 3 GPx enzymes with regard to its substrate specificity and localization. GPx4 has the unique ability to reduce membrane lipid hydroperoxides such as phospholipids and cholesterol hydroperoxides directly, and it is located mostly in the testis [4]. Peroxiredoxins are hydrogen peroxide-scavenging enzymes that were discovered more recently than the above-mentioned catalase and GPx enzymes. Peroxiredoxin enzymes contain conserved cysteine residues that undergo peroxide-dependent oxidation and thiol- (thioredoxin-) dependent reduction during a catalytic cycle. Mammalian cells express 6 isoforms of peroxiredoxin (peroxiredoxins I to VI), which are classified into 3 subgroups (2-Cys, atypical 2-Cys, and 1-Cys) on the basis of the number and position of cysteine residues in the active sites. The relatively high abundance of peroxiredoxin enzymes in mammalian cells appears to be a result of the role these proteins play in removing the low levels of peroxides produced during normal cellular metabolism [5]. Besides the antioxidative enzymes described above, cells have small molecules with antioxidative capacity, such as ascorbic acid and *α*-tocopherol [6], which are able to scavenge ROS efficiently and specifically at relatively low concentrations. When ROS are not eliminated sufficiently by antioxidative enzymes or small molecules, cells are damaged by oxidative insults, leading to cell death.

Steroid hormones synthesized in and secreted from peripheral endocrine glands pass through the blood-brain barrier and perform functions in the central nervous system (CNS). In addition, the brain possesses an inherent endocrine system and synthesizes steroid hormones known as neurosteroids [7]. Several reports have described neurotrophic and neuroprotective properties of steroid hormones, including pregnenolone, pregnenolone sulfate, progesterone, allopregnanolone, dehydroepiandrosterone, dehydroepiandrosterone sulfate, deoxycorticosterone, allotetrahydrodeoxycorticosterone, testosterone, and 17*β*-estradiol [8–14]. The biological functions of the neurosteroids and steroids from circulation are exerted either through a conventional genomic process via estrogen receptors (ERs), androgen receptors, progesterone receptors (PRs), mineral corticoid receptors, and glucocorticoid receptors or through interaction with membrane receptors as allosteric modulators of the gamma-aminobutyric acid (GABA)-A/central-type benzodiazepine receptor complex, N-methyl-d-aspartate receptors, kainate receptors, *α*-amino-3-hydroxy-5-methylisoxazole-4-propionic acid receptors, sigma receptors, glycine receptors, serotonin receptors, nicotinic receptors, and muscarinic receptors. Neurosteroids may directly activate G protein coupled transmembrane receptors or indirectly modulate the binding of neuropeptides to their receptors [15]. Detailed protective mechanisms of steroid hormones are now emerging, and antioxidative action is considered to be a key part of the neuroprotective effects of steroids.

While the protective actions of steroid hormones in the brain under pathophysiological conditions such as Alzheimer's disease and stroke are well studied, the effects of steroids on exogenous neurotoxicants such as environmental chemicals and pesticides are unclear. We have investigated the antioxidative action of neuroactive steroids on organometal-induced toxicity in the brain. In this review, we summarize the antioxidative effects of neuroactive steroids, especially 17*β*-estradiol and progesterone, on neuronal injury in the CNS during various pathological conditions and then describe our recent findings on the neuroprotective effects of 17*β*-estradiol and progesterone on oxidative neuronal injury induced by organometallic compounds, tributyltin, and methylmercury.

## 2. 17**β**-Estradiol Protection against Oxidative Injury in the CNS

17*β*-Estradiol is a female sex steroid that has been shown to have several beneficial effects, such as preventing bone loss [16] and decreasing risk of coronary disease [17], as well as essential functions on female reproduction. 17*β*-Estradiol produces neuroprotective effects during ischemic brain injury [18] and Alzheimer's disease [19]. In addition, 17*β*-estradiol is known to attenuate oxidative stress in a manner that protects the CNS from several harmful stimuli. Treatment of ozone-exposed rats with 17*β*-estradiol suppressed lipid peroxidation and subsequent decreases in cells in the olfactory bulb [20]. 17*β*-Estradiol attenuated ROS levels and behavior disorder induced by irradiation of X-rays in neonatal rats [21]. Oxidative stress and subsequent neurotoxicity induced by ethanol [22], 6-hydroxydopamine [23], and oxygen-glucose deprivation [24] were suppressed by treatment with 17*β*-estradiol. Some antioxidative mechanisms of 17*β*-estradiol have been proposed.

### 2.1. Genomic Pathway of ERs

The intracellular targets of 17*β*-estradiol are ER*α* and ER*β*, which are dominantly located in the nuclei. ERs are classified as a nuclear receptor superfamily and form the transcription initiation complex on the estrogen response element, followed by transcription of target genes, regulating several cellular functions (the genomic pathway of ERs). Regulation of gene expression by 17*β*-estradiol is closely related to its antioxidative action. Upregulation of antioxidative enzymes is the simplest of the antioxidative mechanisms of 17*β*-estradiol that are mediated via the genomic ER pathway. SK-N-MC human neuroblastoma cells with ER*α* overexpression showed increased expression of GPx and catalase [25]. Therefore, ER*α* is thought to confer cellular tolerance against oxidative stress. In contrast, overexpression of ER*β* did not affect the expression of GPx and catalase [25]. In addition, 17*β*-estradiol pretreatment decreases infarct volume in ER*β* null mice, but not in ER*α* null mice, that have been subjected to middle cerebral artery occlusion, suggesting that ER*α*, rather than ER*β*, is centrally involved in 17*β*-estradiol-mediated neuroprotection against oxidative stress [26, 27]. Administration of 17*β*-estradiol increased SOD1 and SOD2 immunoreactivity in nigral neurons of mice subjected to the 1-methyl-4-phenyl-1,2,3,6-tetrahydropyridine treatment method of Parkinson's disease induction [28]. 17*β*-Estradiol treatment for 11 days also reduced nitrotyrosine immunoreactivity in nigral neurons, suggesting that 17*β*-estradiol suppressed oxidative stress via SOD1 and SOD2 upregulation. Oxidative stress and neuronal cell death induced by oxygen-glucose deprivation in mouse brain slices were attenuated by 17*β*-estradiol treatment via upregulation of SOD1 [24]. Thus, SOD seems to be a major target of 17*β*-estradiol to attenuate oxidative neuronal injury via the genomic ER pathway. Superoxide is initially generated in organisms, and it is converted to hydrogen peroxide, hydroxyl radicals, and other reactive metabolites [29]. Because SOD also suppresses the production of hydrogen peroxide and hydroxyl radicals by eliminating superoxide, upregulation of SOD by 17*β*-estradiol might produce antioxidative responses in living animals. Phase II antioxidative enzymes, glutathione S-transferase (GST) and NADPH:quinone oxidoreductase, are reported to be induced in the rat brain by chronic exposure to 17*β*-estradiol [30], indicating that several antioxidative enzymes other than SOD also might be targets of genomic ER signals.

SK-N-MC cells with ER*α* overexpression also showed reduced expression of neuronal nitric oxide synthase and inducible nitric oxide synthase [25]. Superoxide and nitric oxide are readily converted by nonenzymic chemical reactions into reactive nonradical species peroxynitrite (ONOO^−^), which can in turn give rise to new radicals, leading to cellular injury. Therefore, decreases in nitric oxide synthase might contribute to suppress oxidative stress via elimination of reactive radical species.

Factors with protective effects in the CNS in addition to antioxidative enzymes induced by 17*β*-estradiol have been reported. Seladin-1 was found to be downregulated in brain regions affected by Alzheimer's disease [31]. Thereafter, seladin-1 was demonstrated to encode the gene 3*β*-hydroxysterol Δ24-reductase, which catalyzes the reduction of the Δ24 double bond of desmosterol to generate cholesterol [32]. 17*β*-Estradiol upregulates seladin-1 in long-term neuroblast cell cultures from human fetal olfactory epithelium [33], and increased seladin-1 via ER*α*-dependent transcription protects neurons from *β*-amyloid and hydrogen peroxide toxicity by suppressing caspase-3 activity and oxidative stress [33, 34]. The promoter region of seladin-1 includes the estrogen responsive element [34]. Neuroglobin is also a target of 17*β*-estradiol in the brain. Neuroglobin was initially discovered in neurons as a 150 amino acid-long heme-protein displaying <25% sequence identity to conventional vertebrate hemoglobin or myoglobin [35, 36], and neuroglobin was shown to protect neuron from several neurotoxic conditions such as ischemia in vivo and in vitro [37–39]. Recently, 17*β*-estradiol was reported to upregulate neuroglobin via ER*β* in the SK-N-BE human neuroblastoma cell line, and this effect inhibited apoptosis induced by hydrogen peroxide [40]. In addition, neuroglobin elicited by 17*β*-estradiol decreased inflammatory markers interleukin 6 and interferon *γ*-inducible protein 10 in primary astrocytes [41]. Moreover, proapoptotic genes are suppressed by treatment with 17*β*-estradiol. Hydrogen peroxide induced apoptosis in the C6 rat glioma cell line, with upregulation of proapoptotic Bcl family protein Bax [42], and 17*β*-estradiol attenuated Bax upregulation and hydrogen peroxide-induced apoptosis [42]. Because C6 cells express ER*β*, but not ER*α*, this protective action of 17*β*-estradiol might be due to ER*β*-dependent signaling [42]. 17*β*-Estradiol regulates numerous genes directly involved in or likely related to the modulation of oxidative stress via the genomic ER pathway. Multiple factors, such as cell species, stimuli harmful to the CNS, ER expression levels, and cofactors binding to the ER, might interfere with the antioxidative effects caused by transcriptional regulation by 17*β*-estradiol. Genomic actions of 17*β*-estradiol are listed in Table 1.

### 2.2. Nongenomic Pathway of ERs

ERs are also located in the cytosol, and stimulation of cytosolic ERs elicits intracellular signaling events such as kinase activation (the nongenomic pathway of ERs). These signals protect neurons from toxic stimuli, similar to the regulation of gene expression mediated by ERs. It is well established that 17*β*-estradiol activates extracellular signal-regulated kinase (ERK), a mitogen-activated protein kinase (MAPK). Dorsa and colleagues demonstrated that 17*β*-estradiol induced ERK phosphorylation via an ER*α*- and ER*β*-dependent nongenomic pathway using the HT22 hippocampus-derived cell line, which overexpresses ER*α* and ER*β* [43, 44]. The protective effects of 17*β*-estradiol against oxidative cell death induced by amyloid *β* or glutamate were largely attenuated by pretreatment with the MEK inhibitor PD98059 [43, 44], clearly indicating that ERK activation by 17*β*-estradiol via the nongenomic ER pathway is involved in neuroprotection against oxidative injury. Akt is also activated downstream of ERs. Zhang et al. reported that extranuclear ER*α* stimulated by 17*β*-estradiol induced Akt phosphorylation and subsequent phosphorylation/inactivation of Rac1, a factor critical for activation of NOX2 NADPH oxidase, decreasing superoxide generation from NOX2 and attenuating oxidative neuronal damage elicited by ischemia in the hippocampal CA1 region [45]. In addition, neuroprotective effects against amyloid *β* toxicity are thought to be involved in the formation of the ER*α*/GSK-3*β*/*β*-catenin complex, resulting in modulation of Wnt signaling [46]. According to this hypothesis, ER*α* can regulate intracellular Ca^2+^ levels independent of its transcriptional activity.

### 2.3. Mitochondrial Efficiency

Several lines of evidence support the possibility that 17*β*-estradiol exerts its potent neuroprotective effects through a mitochondrial mechanism. Mitochondria are major sites of ROS generation inside cells. Approximately 1 to 5% of the total oxygen consumed in the mitochondrial respiratory chain is incompletely reduced and thus leads to ROS production [47, 48]. It is generally accepted that superoxide generated from the respiratory complex in the inner mitochondrial membrane is vectorially released into the mitochondrial matrix and subsequently dismutated to hydrogen peroxide by SOD2, although the direct release of superoxide into the intermembrane space has been proposed [49]. Brain mitochondria isolated from rats treated with 17*β*-estradiol showed increased expression and activity of the electron transport chain complex IV (cytochrome c oxidase) [50], and this change was coupled with a decreased rate of ROS leakage and reduced lipid peroxidation, representing a systematic enhancement of brain mitochondrial efficiency. Mitochondria isolated from cerebral blood vessels obtained from ovariectomized rats with 17*β*-estradiol replacement also showed increased complex IV activity and decreased hydrogen peroxide production from mitochondria [51]. 17*β*-Estradiol increased levels of cardiolipin, and thus restored mitochondrial integrity [52]. In brain endothelial cells, 17*β*-estradiol treatment reduced ROS derived from mitochondria due to increased cytochrome c, but not ROS elimination by SOD2 [53]. In rat primary astrocytes, 17*β*-estradiol attenuated ROS generation, ATP depletion, and mitochondrial membrane potential decreases accompanied by oxygen-glucose deprivation and subsequent reoxygenation [54].

ER*β* is reported to be localized to mitochondria in rat primary neurons [55]. ER*β* knockdown results in a lower resting mitochondrial membrane potential, and these cells show the resistance to oxidative stress-induced depolarization of mitochondrial membrane potential, ATP depletion, and ROS generation [56], suggesting ER*β* in mitochondria could function as a mitochondrial vulnerability factor that is involved in mitochondrial membrane potential maintenance and mitochondrial vulnerability. In addition, mitochondrial ER*β* is reduced in female Alzheimer's disease patients [57]. Higher ROS generation in brain mitochondria is observed in ER*β*-knockout mice treated with amyloid beta-peptides compared with wild-type mice. Taken together, these results show that 17*β*-estradiol could induce mitochondrial alterations in the CNS in a manner that reduces oxidative stress.

### 2.4. Direct Elimination of ROS

Antioxidant effects have been proposed as a mechanism through which the neuroprotective action of 17*β*-estradiol is mediated. Behl et al. reported that high concentrations (micromolar) of 17*β*-estradiol or 17*β*-estradiol, an isomer of 17*β*-estradiol that is at least 200-fold less active as a hormone [58], showed neuroprotective effects against oxidative stress. Estrogen and estrogen derivatives with a hydroxyl group at the C3 position on the A-ring can act as powerful neuroprotectants in a short-term, ER-independent manner because of their antioxidant capacity [58]. The concentrations of 17*β*-estradiol required for significant antioxidative neuroprotection by direct radical scavenging activity tend to be higher than the estrogen levels that occur naturally in vivo [59, 60]. However, remarkably, a mechanism through which relatively low concentrations of 17*β*-estradiol effectively and directly scavenge radicals was proposed using a synthetic derivative of 17*β*-estradiol, 17*β*-butoxy-1,3,5(10)-estratrien-3-ol, via a radical-scavenging antioxidant cycle catalyzed by reductases [61]. The phenolic A-ring is transformed into 10*β*-butoxy-17*β*-butoxy-1,3,5(10)-estratrien-3-one, a nonaromatic paraquinol, upon capturing hydroxyl radicals, which results in the complete loss of ER affinity and antioxidant activity, after which the parent compound is recovered from paraquinol via enzyme-catalyzed NADPH-dependent reductive aromatization without causing oxidative stress. In this reaction, 17*β*-estradiol could enzymatically eliminate hydroxyl radicals with NADPH consumption. This process may explain how 17*β*-estradiol acts as an antioxidant and attenuates oxidative stress at low concentrations. However, a quinone-quinol redox cycle, which is also catalyzed by NADPH-dependent reductases, is known to generate superoxide, leading to oxidative cell injury [62]. Because there are numerous endogenous quinone compounds, such as dopamine quinones, in the brain, the efficiency of the 17*β*-estradiol antioxidant cycle might depend on reductase activity, as well as reductase specificity for 17*β*-estradiol-derived paraquinol.

### 2.5. Microglial Inactivation

Microglia, a type of glial cell in the CNS, are target cells through which 17*β*-estradiol protects neurons from excess inflammation. Microglia are the primary immune cells of the CNS and are activated quickly in response to external pathogens or cell debris, after which they act by releasing inflammatory factors or engulfing foreign bodies to mediate the inflammatory response. However, excessive activation of microglia may be harmful for host cells; microglia can promote the development of some neuronal diseases by producing large amounts of cytokines and other inflammatory molecules such as tumor necrosis factor-*α*, interleukin-1*β*, nitric oxide, and ROS. Activated microglia are associated with the pathogenesis of Parkinson's disease, Alzheimer's disease, ischemia-reperfusion injury, trauma, epilepsy, depression, and schizophrenia [63–67].

Recently, increasing evidence has shown that 17*β*-estradiol protects neurons from excess or prolonged inflammation in the brain. We demonstrated that rat primary microglia express both ER*α* and ER*β* predominantly in the nucleus [68]; therefore, microglia can respond to 17*β*-estradiol stimulation. Treatment with 17*β*-estradiol suppresses inflammatory cytokine expression and nitric oxide production induced by lipopolysaccharide in microglia [69, 70]. These suppressive effects of 17*β*-estradiol are mediated via the ERs and act by blocking DNA binding and transcriptional activation by nuclear factor-kappa B p65 by preventing its nuclear translocation [71]. 17*β*-Estradiol has also been reported to inhibit neuroinflammation in an ER-dependent manner in studies using in vivo models of CNS diseases [72, 73]. Therefore, the antioxidative activity of 17*β*-estradiol could be mediated by suppressive effects on activated microglia.

### 2.6. Other Mechanisms

**Figure 1:**
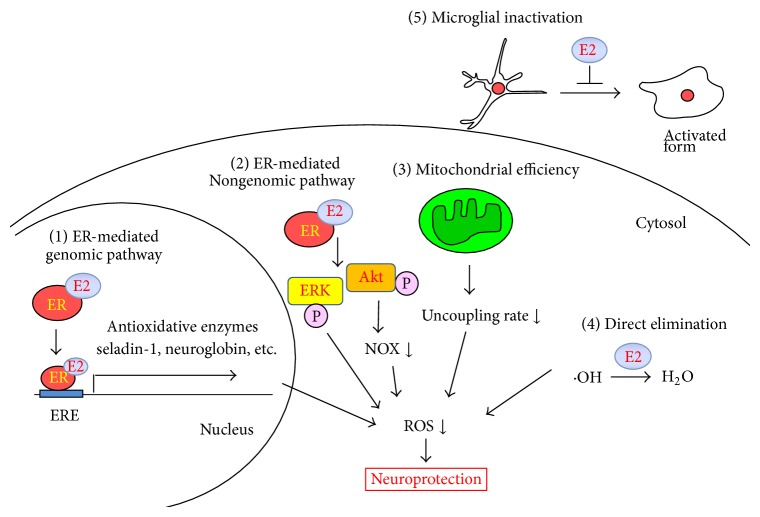
Antioxidative mechanisms of 17*β*-estradiol in the brain. The proposed antioxidative mechanisms of 17*β*-estradiol in the CNS are as follows: (1) Antioxidative enzymes or other functional proteins are transcriptionally activated by ER signaling. (2) Intracellular survival signaling is activated by ER independent of transcriptional regulation. (3) 17*β*-Estradiol affects mitochondrial antioxidative enzymes or respiratory complexes to decrease ROS by enhancing mitochondrial efficiency. (4) 17*β*-Estradiol directly scavenges ROS or other reactive radicals. (5) 17*β*-Estradiol suppresses ROS generation from microglia by inhibiting their activation. E2, 17*β*-estradiol; ER, estrogen receptor; ERE, estrogen response element; ERK, extracellular signal-regulated kinase; NOX, NADPH oxidase; ROS, reactive oxygen species.

Because the antioxidative effects of 17*β*-estradiol are mediated by the diverse mechanisms described above, they may be the sum of both ER-dependent and ER-independent actions. In addition, Simpkins and colleagues demonstrated that 17*β*-estradiol attenuated decreases in the activity of serine/threonine protein phosphatases PP1, PP2A, and calcineurin induced by glutamate excitotoxicity in rat primary neurons [74] and middle cerebral artery occlusion in rats [75] and thus suppressed oxidative neuronal injury. ERs are not involved in these protective effects of 17*β*-estradiol. 17*β*-Estradiol likely protects cells by blocking ubiquitination and/or degradation of protein phosphatases caused by oxidative or excitotoxic stress. Therefore, novel targets might be also fundamental for 17*β*-estradiol-mediated neuroprotection against oxidative stress. The antioxidative effects of 17*β*-estradiol are presented schematically in Figure 1.

The expression of cytochrome P450 aromatase (CYP19), which synthesizes 17*β*-estradiol from testosterone, is increased by oxidative stress in neurons and astrocytes [76, 77], indicating that oxidative stress could potentiate 17*β*-estradiol production. Cultured astrocytes isolated from female rats with high expression levels of cytochrome P450 aromatase showed better tolerance to cellular injury induced by oxygen-glucose deprivation than male rats with low cytochrome P450 aromatase expression [78]. Moreover, sex differences in oxygen-glucose deprivation-induced cell death were diminished by inhibition of cytochrome P450 aromatase in astrocytes prepared from female rats. These data indicate that expression of cytochrome P450 aromatase is a determinant for the severity of neuronal injury, and 17*β*-estradiol might play a role in adaptive responses against oxidative stress in the brain.

Several negative effects of 17*β*-estradiol on oxidative stress have been reported. 17*β*-Estradiol did not suppress glutamate excitotoxicity and apoptosis mediated by potassium-deprivation or ceramide-mediated apoptosis in cerebellar granule cells [79]. Because 17*β*-estradiol activated ERK in these studies, the authors concluded that ERK activation induced by 17*β*-estradiol treatment was not involved in its neuroprotective effects. Furthermore, Gordon et al. reported that administration of 17*β*-estradiol exacerbated infarct formation and lipid peroxidation induced by middle cerebral artery occlusion [80]. Chronic 17*β*-estradiol treatment reportedly enhanced expression of glial fibrillary acidic protein and interleukin-1*β* in the arcuate nucleus, followed by potentiation of oxidative stress [81]. In this regard, the effects of 17*β*-estradiol on oxidative stress are suggested to be tissue-specific and dependent on the timing of the treatment. Circulating estrogens are negatively correlated with ER*α* expression in the inner ear [82], indicating that 17*β*-estradiol could downregulate ERs in certain situations. Careful consideration of ER dynamics might be required for effective evaluation of the effects of 17*β*-estradiol on oxidative stress.

## 3. Progesterone Protection against Oxidative Neuronal Injury

Similar to 17*β*-estradiol, progesterone is a female sex steroid that is present in the brain; however, there are far fewer reports on the antioxidative action of progesterone. Progesterone suppresses impairment of memory and oxidative stress, lipid peroxidation, and protein oxidation, induced by phosphamidon, an organophosphorus pesticide, in rats [83]. Progesterone suppressed lipid peroxidation and decreases in GSH levels caused by brain ischemia-reperfusion injury in rats [84]. Progesterone binds to the PR in the nucleus, activating gene transcription. SOD, GPx, catalase, and glutathione reductase are target genes of progesterone involved in oxidative stress tolerance [85–87]. Therefore, progesterone protects neuronal cells from oxidative stress by upregulating antioxidative enzymes via a PR-dependent genomic pathway. In addition, progesterone reportedly decreased mitochondrial ROS production by upregulating complex IV and SOD2 expression, followed by increased respiratory activity [50]. Thus, the antioxidative effects of progesterone are similar, at least in part, to those of 17*β*-estradiol.

Negative effects of progesterone on oxidative stress are also reported. Progesterone does not alter ischemic oxidative stress in rats subjected to a decapitation ischemia model [88]. Furthermore, the clinical progestin medroxyprogesterone is reported to counteract the antioxidative effects of 17*β*-estradiol, such as increased expressions of SOD2 and peroxiredoxin V, decreases in lipid peroxides, and enhancement of mitochondrial respiration in primary cultures of hippocampal neurons and glial cells [89]. Further study is needed to reveal the overall antioxidative actions of progesterone, and the manner in which it might antagonize the effects of 17*β*-estradiol.

## 4. Oxidative Stress and Neurotoxicity Induced by Tributyltin (TBT)

Organotin compounds have long been used as thermal stabilizers, catalytic agents, and biocidal compounds for preserving wood, textiles, cordage fibers, and electronic equipment [90]. Among organotin compounds, TBT has been most widely used in paint formulations to prevent marine fouling on ships, boats, and fish nets. However, concerns about toxic effects such as imposex in sea snails and other malformations in marine organisms led to a ban on the use of TBT in antifouling compounds [91]. Nonetheless, the pollution of coastal waters continues. Environmental surveying and monitoring of TBT are conducted in order to prevent the consumption of bioaccumulated TBT by humans. The average intake of TBT by humans from market-bought seafood has been estimated to vary worldwide between 0.18 and 2.6 *μ*g per day per person [92], and butyltin compounds, including TBT, have been reported at concentrations between 50 nM and 400 nM in human blood [93]. TBT is thought to be metabolized successively to dibutyltin (DBT) and monobutyltin (MBT). However, an examination of butyltin compounds in human blood collected from subjects in central Michigan revealed that the concentration of TBT in blood was almost the same as that of DBT and MBT [94]. Therefore, a certain quantity of TBT remains in blood without metabolism. TBT are highly lipophilic and readily penetrate the blood-brain barrier to enter the brain. Indeed, the administration of TBT elicited abnormal behavior and reduced brain weight within the cerebellum and synaptogenesis in rats [95–98]. Therefore, the effects of TBT on the CNS are of great concern.

Neural cell death induced by TBT in rat hippocampal slices was reportedly suppressed by pretreatment with antioxidants, including SOD, catalase, trolox, and tocopherol [99]. Furthermore, Kurita et al. demonstrated that hydrogen peroxide was generated by treatment of rat cortical neurons with TBT [100]. Therefore, induction of oxidative stress is considered to be one of the earlier events in the process of TBT-induced neuronal cell death. We also demonstrated that TBT induced ROS production and lipid peroxidation in rat hippocampal slices [101]. However, few reports have examined oxidative injury induced by TBT in detail, and there is little evidence of the mechanism through which TBT elicits ROS production. Recently, we reported that TBT strongly inhibited GST in the hippocampus, but we did not find effects on other GSH-related enzymes, including GPx and glutathione reductase [101]. While pretreatment with ethacrynic acid, a potent GST inhibitor, potentiates ROS production, lipid peroxidation, and neuronal cell death induced by TBT, pretreatment with sulforaphane, which induces GST expression in hippocampal slices, largely suppresses TBT-elicited oxidative stress and cell death [101]. These results indicate that GST inhibition by TBT is a potent mechanism through which it generates ROS and oxidative neuronal injury. GST is a phase II-detoxifying enzyme that conjugates GSH to xenobiotic and endogenous toxins to facilitate their excretion from the organism [102]. Substrates for GST inside cells are organic hydroperoxides and 4-hydroxyalkenals [103], which can induce oxidative stress [104–107]. Therefore, TBT may induce oxidative stress by suppressing degradation of oxidative metabolites, leading to GST inhibition. Furthermore, GST metabolizes cyclized o-quinones, which are oxidized products of catecholamines, via GSH conjugation [108, 109]. When intracellular o-quinones are not metabolized, they undergo one-electron reduction by intracellular reductases to yield semiquinone radicals, which are then reoxidized and can enter redox cycles in cooperation with molecular oxygen to form superoxide anions [62]. Therefore, TBT might induce oxidative stress via potentiation of the quinone redox cycle.

## 5. Protective Effects of 17**β**-Estradiol and Progesterone on TBT-Induced Neuronal Injury

**Figure 2:**
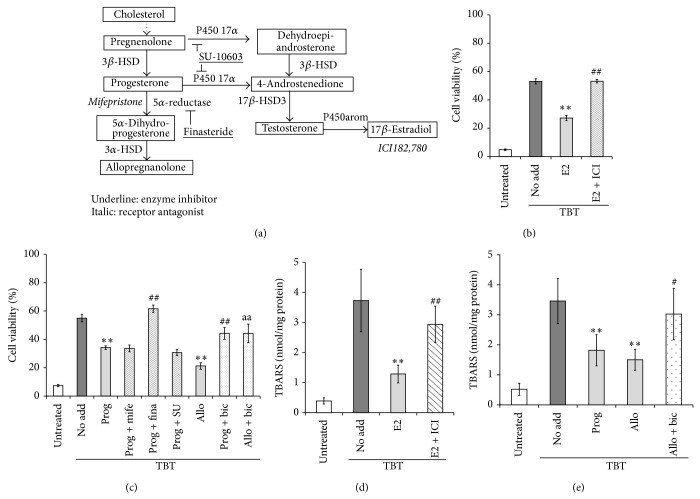
Effects of 17*β*-estradiol and progesterone on TBT-induced oxidative stress and cell death in rat hippocampal slices. (a) Pathway of steroid hormone metabolism in the rat hippocampus. HSD, hydroxysteroid dehydrogenase. (b, d) Rat hippocampal slices were pretreated with ER antagonist ICI182,780 (ICI, 100 *μ*M) for 20 min, after which 1 *μ*M of 17*β*-estradiol (E2) was added to the culture. After 6 h of incubation, the slices were treated with 3 *μ*M of TBT for 24 h. Cell viability of the CA1, CA3, and dentate gyrus regions was measured by propidium iodide staining (b), and lipid peroxidation was evaluated by determination of thiobarbituric acid reactive substance (TBARS) content (d). The reported values are the mean ± S.E. of 5 separate experiments. ^**^ *P* < 0.01 versus 3 *μ*M TBT-treated group. ^##^ *P* < 0.01 versus 3 *μ*M TBT and 1 *μ*M 17*β*-estradiol-treated group. (c, e) Rat hippocampal slices were pretreated with progesterone receptor antagonist mifepristone (Mife, 10 *μ*M), 5*α*-reductase inhibitor finasteride (Fina, 100 *μ*M), cytochrome P450 17*α* inhibitor SU-10603 (SU, 20 *μ*M), or GABA_A_ receptor antagonist bicuculline (Bic, 100 *μ*M), after which progesterone (Prog, 1 *μ*M) or allopregnanolone (Allo, 1 *μ*M) was added to the culture. After a 2 h incubation, the slices were exposed to 3 *μ*M TBT for 24 h. Cell viability (c) and lipid peroxidation (e) were evaluated. The reported values are the mean ± S.E. of 5 separate experiments. ^**^ *P* < 0.01 versus 3 *μ*M TBT-treated group. ^#^ *P* < 0.05, ^##^ *P* < 0.01 versus 3 *μ*M TBT and 1 *μ*M progesterone-treated group. ^aa^ *P* < 0.01 versus 3 *μ*M TBT and 1 *μ*M allopregnanolone-treated group.

Steroid hormones synthesized in and secreted from peripheral endocrine glands pass through the blood-brain barrier and perform functions in the CNS. In addition, the brain possesses an inherent endocrine system and synthesizes some steroid hormones [110]. The hippocampus can actively synthesize steroid hormones because steroid hormone synthesizing enzymes are highly expressed (Figure 2(a)). Therefore, we investigated whether two female sex steroids, 17*β*-estradiol and progesterone, prevent neuronal oxidative injury induced by TBT using rat organotypic hippocampal slice cultures.

In our study, pretreatment with 17*β*-estradiol dose-dependently suppressed ROS production, lipid peroxidation, and neuronal cell death elicited by TBT (Figures 2(b) and 2(d), and [111]). ICI182,780, an ER antagonist, abolished neuroprotection mediated by 17*β*-estradiol, but actinomycin D and cycloheximide, an mRNA synthesis inhibitor and a protein synthesis inhibitor, respectively, did not show any effects on decreases in cell viability induced by TBT [111], clearly indicating that neuroprotection by 17*β*-estradiol against TBT-induced neuronal injury is mediated by the nongenomic ER-dependent signaling pathway. Because Akt was activated by treatment with 17*β*-estradiol and the protective effects of 17*β*-estradiol on TBT-induced oxidative stress and subsequent cell death were attenuated by the Akt inhibitor triciribine [111], Akt might be responsible for suppressing TBT-mediated neuronal oxidative injury downstream of the ER in a manner independent of gene transcription. Once generated, ROS present anywhere inside cells trigger ROS release by mitochondria (ROS-induced ROS release), amplifying oxidative stress [112]; thus, mitochondria are the major source and target of ROS. 17*β*-Estradiol suppresses hydrogen peroxide-induced apoptosis and protects mitochondria from ROS by phosphorylating Akt, and subsequently Bad, via the ER*α* and ER*β*-dependent pathway [113]. Therefore, the protective effect of Akt activation on mitochondria may contribute to the attenuation of TBT-induced neuronal injury by 17*β*-estradiol. Akt attenuates ROS production by decreasing NADPH oxidase activity following Rac1 phosphorylation [45]. In addition, a GST inhibitor was reported to decrease Akt activity [114]. We also reported that TBT induces oxidative stress via GST inhibition [101]. Considering these findings, a reduction in NADPH oxidase activity may be involved in the suppression of oxidative stress mediated by 17*β*-estradiol.

Progesterone also showed protective effects against TBT-induced neuronal injury. Pretreatment of rat hippocampal slices with progesterone significantly attenuated lipid peroxidation and cell death induced by TBT (Figures 2(c) and 2(e), and [115]). Interestingly, mifepristone, a progesterone receptor antagonist, did not affect neuronal cell death induced by TBT, indicating that the protective effects of progesterone were not mediated by activation of the progesterone receptor [115]. Alternatively, allopregnanolone, a reactive metabolite of progesterone, is thought to mediate neuroprotection against TBT-elicited oxidative neuronal injury for the following 3 reasons: (i) progesterone added in the culture is converted to allopregnanolone, (ii) inhibition of the metabolism of progesterone to allopregnanolone by the 5*α*-reductase inhibitor finasteride abolished the neuroprotective action of progesterone, and (iii) pretreatment of hippocampal slices with allopregnanolone largely suppressed lipid peroxidation and cell death induced by TBT (Figures 2(c) and 2(e), and [115]). Increasing evidence shows that allopregnanolone acts as an agonist of GABA_A_ receptors [116], and the neuroprotective effect of allopregnanolone was revealed to be dependent on GABA_A_ receptor activity [117]. In our study, pretreatment with bicuculline, a potent GABA_A_ receptor antagonist, significantly abrogated the neuroprotective actions of progesterone and allopregnanolone (Figures 2(c) and 2(e), and [115]), indicating that the GABA_A_ receptor is involved in the protective effects of progesterone on neuronal injury induced by TBT. Nakatsu et al. showed that TBT-stimulated potentiation of glutamate release in rat cortical neurons occurred upstream of ROS generation [118]. Furthermore, the GABA_A_ receptor agonist muscimol attenuated glutamate release and subsequent ROS production and thus suppressed cell death induced by amyloid *β* proteins (25–35) in rat cortical neurons [119]. Therefore, in our experimental system, the neuroprotective effects of progesterone, for example, attenuation of TBT-induced excitotoxicity and subsequent oxidative injury, could be mediated by GABA_A_ receptor activation. However, the protective effects of progesterone and allopregnanolone against TBT in hippocampal neurons were only partial, indicating the diverse mechanisms of cell death induced by TBT.

**Figure 3:**
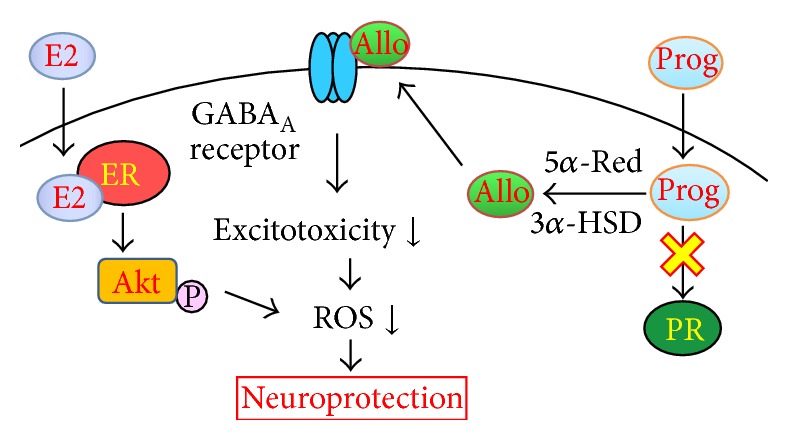
Putative antioxidative mechanisms of 17*β*-estradiol and progesterone in TBT-induced oxidative neuronal injury. 17*β*-Estradiol (E2) suppresses TBT-induced neuronal injury via an ER-dependent nongenomic pathway. The attenuation of oxidative stress downstream of Akt activation is considered to be involved in the neuroprotection mediated by 17*β*-estradiol. Progesterone is readily converted to allopregnanolone, and the neuroprotective activity of allopregnanolone is attributed to modulation of GABA_A_ receptor activity. TBT-induced oxidative stress could be suppressed by multiple pathways stimulated by neuroactive steroids. Allo, allopregnanolone; E2, 17*β*-estradiol; ER, estrogen receptor; 3*α*-HSD, 3*α*-hydroxysteroid dehydrogenase; Prog, progesterone; PR, progesterone receptor; 5*α*-red, 5*α*-reductase; ROS, reactive oxygen species.

TBT can induce oxidative stress through various mechanisms such as GST inhibition and glutamate excitotoxicity. However, interestingly, multiple neuroactive steroids partly transconverted in hippocampal slices can suppress oxidative stress via several pathways (Figure 3). Therefore, neuroactive steroids might protect against xenobiotics and, considering the constant synthesis of steroid hormones in the brain, might be involved in adaptive reactions to xenobiotics. In the next section, we discuss the action of de novo synthesized steroid hormones on oxidative neuronal injury in the hippocampus.

## 6. Oxidative Stress Induced by Methylmercury (MeHg)

MeHg is a hazardous pollutant to which humans are exposed mainly through consumption of fish [120, 121]. Minamata disease, anthropogenic exposure to MeHg in Japan, and MeHg poisoning in Iraq have established the toxicity of MeHg in the nervous system [122–124]. Once MeHg is taken into the blood via food intake, it easily passes through the blood-brain barrier as a cysteine conjugate by using the neutral amino acid transport system and thus quickly reaches the brain [125, 126]. Thus, the primary target of MeHg is the CNS, and MeHg produces abnormal behaviors via CNS disruption [123]. Indeed, MeHg elicits sensory and auditory impairment in humans [127], visual disturbances and tremors in cats and monkeys [128, 129], and hind limb bending in rats [130].

Oxidative stress is a major toxic mechanism of MeHg in the CNS (reviewed in [131]). MeHg can interact with nucleophilic groups, mainly thiol and selenol, because of its electrophilicity. Indeed, MeHg reacts with R-SH and R-SeH to form the very stable complexes R-SHgCH_3_ and R-SeHgCH_3_. Considering that many metabolic enzymes and antioxidative enzymes include thiol and/or selenol groups through which their catalytic activity can be regulated and that MeHg affects the activity of such enzymes [132–136], these interactions could induce imbalances between ROS and cellular antioxidative capacity, leading to oxidative cell damage.

GPx, which includes selenol in its active site, is an intracellular target of MeHg. Due to the high affinity of MeHg for selenol groups, decreased GPx activity after MeHg exposure has been attributed to direct inhibition of the enzyme [137]. Furthermore, MeHg has been reported to induce a selenium-deficient-like condition [138]. Thus, MeHg can be considered to cause decreases in GPx synthesis. Reduction of GPx activity inside cells could increase levels of ROS or related reactive compounds such as hydrogen peroxide and lipid peroxide, leading to neuronal oxidative injury. MeHg also acts on mitochondria. MeHg stimulates superoxide production independent of the effects against SOD2 [139, 140]. Complexes II and III in the respiratory chain are reported to be targets of MeHg, and impairment of these complexes elicits excess production of hydrogen peroxide [141]. In addition, MeHg induces glutamate dyshomeostasis. MeHg has been shown to inhibit glutamate uptake into cultured astrocytes [142, 143], rat synaptic vesicles [144], and cerebral cortical slices [145] and to increase spontaneous release of glutamate from mouse cerebellar slices [146] and cultured neuronal cells [147], indicating that MeHg may induce increases in extracellular glutamate levels. These in vitro findings have been confirmed in vivo with microdialysis probes implanted in the frontal cortex of adult rats [148]. Therefore, MeHg can elicit oxidative stress via glutamate excitotoxicity.

Some endogenous molecules reportedly attenuate MeHg toxicity. In MeHg-treated rodents, treatment with vitamin E and selenium suppresses decreases in body weight and neurological symptoms such as auditory responses [149–151]. Vitamin K, pyrroloquinoline quinone, and metallothionein also attenuate cytotoxicity induced by MeHg [152–155]. In addition, MeHg activates the transcription factor Nrf-2 in a manner coupled to S-mercuration of its negative regulator Keap1, and the Keap1/Nrf-2 pathway protects neurons from MeHg-induced oxidative injury [156]. Together, the balance between ROS production induced by MeHg and endogenous and/or inducible protective mechanisms that act against MeHg might determine levels of oxidative stress and cellular damage.

## 7. Protective Effects of De Novo Synthesized 17**β**-Estradiol on MeHg-Induced Neuronal Injury

The toxic effect of MeHg has been shown to be attenuated by exogenous 17*β*-estradiol in primary cultured rat cerebellar granule cells. In these cultured cells, 17*β*-estradiol protected against MeHg toxicity by acting as an antioxidant without stimulating ERs [157]. In male mice, 17*β*-estradiol administration partially prevented MeHg-induced motor activity deficits and modification of cerebellar glutathione metabolism [158]. These data indicate that 17*β*-estradiol suppresses oxidative stress and subsequent cellular injury induced by MeHg. As described in the Introduction, the brain synthesizes some steroid hormones, including 17*β*-estradiol, and we have previously shown that cultured hippocampal slices also produce 17*β*-estradiol [159]. Thus, we examined the protective action of de novo synthesized 17*β*-estradiol against MeHg-induced cell death.

**Figure 4:**
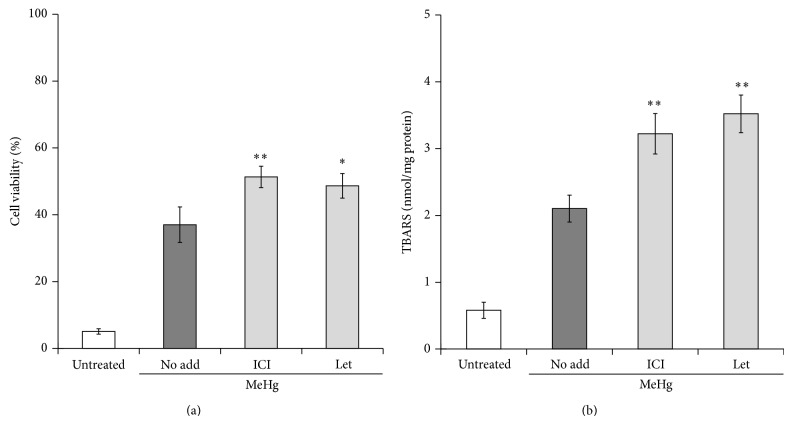
Suppressive effects of de novo synthesized 17*β*-estradiol on MeHg-induced oxidative stress and cell death in rat hippocampal slices. Rat hippocampal slices were pretreated with ER antagonist ICI182,780 (ICI, 100 *μ*M) or cytochrome P450 aromatase inhibitor letrozole (Let, 10 *μ*M) for 20 min and then the slices were treated with 1 *μ*M of MeHg for 24 h. Cell viability of the dentate gyrus region was measured by propidium iodide staining (a), and lipid peroxidation was evaluated by determination of thiobarbituric acid reactive substance (TBARS) contents (b). The reported values are the mean ± S.E. of 5 separate experiments. ^*^ *P* < 0.05, ^**^ *P* < 0.01 versus 1 *μ*M MeHg-treated group.

MeHg exposure injured hippocampal neurons, especially in the dentate gyrus region [160]. Exogenous treatment with 17*β*-estradiol clearly suppressed MeHg-induced neuronal cell death, and this protective action was abolished by the ER antagonist ICI182,780 [160], showing that 17*β*-estradiol protects neurons from MeHg-toxicity in an ER-dependent manner. Interestingly, MeHg neurotoxicity was enhanced by pretreatment with ICI182,780 (Figure 4(a) and [160]). Lipid peroxidation induced by MeHg was also potentiated by ICI182,780 (Figure 4(b)). These results suggest that ER activation attenuated MeHg-mediated oxidative neuronal injury in the absence of added 17*β*-estradiol. Treatment of hippocampal slices with letrozole, which inhibits cytochrome P450 aromatase and thus suppresses 17*β*-estradiol synthesis, significantly enhances lipid peroxidation and cell death elicited by MeHg (Figures 4(a) and 4(b) and [160]). Therefore, considering that cultured hippocampal slices synthesized 17*β*-estradiol [159], de novo synthesized 17*β*-estradiol can protect neurons from MeHg-induced oxidative neuronal injury. We have observed that progesterone and allopregnanolone also suppress neuronal injury induced by MeHg in rat hippocampal slices (unpublished data). Thus, various types of neurosteroids might show neuroprotective action against environmental chemicals, including MeHg.

## 8. Conclusion

Few studies have reported the protective effects of 17*β*-estradiol and progesterone on neurotoxicity induced by environmental chemicals. We have demonstrated the protective actions of 17*β*-estradiol and progesterone on organometallic compound-induced oxidative neuronal injury, and other groups have reported that 17*β*-estradiol attenuated oxidative stress and cell death induced by lead [161], suggesting that 17*β*-estradiol and progesterone can act as endogenous protective factors against environmental chemicals. This hypothesis is supported by the finding that steroid hormone levels in the brain at birth, when the blood-brain barrier is immature and thus the brain is exposed to various xenobiotics, are much higher than those in the brain of an adult [162, 163].

The brain is supplied with steroid hormones from the blood and synthesizes steroid hormones (neurosteroids) to be able to respond to stresses. Many environmental chemicals are known to induce oxidative stress, and the brain could increase production of 17*β*-estradiol when it detects oxidative stress. In this regard, neuroactive steroids might mediate adaptive responses in the CNS to environmental chemicals. As described in this review, 17*β*-estradiol and progesterone induce divergent intracellular events in the CNS under oxidative stress. The sum of these events determines the response of cells and/or individuals. To reveal the interactions between neuroactive steroids and environmental chemicals (or other harmful stimuli), the protective mechanisms of steroids must be identified.

## Figures and Tables

**Table 1:** Genes involved in antioxidative neuroprotection mediated by 17*β*-estradiol in the CNS.

Target gene (up or down)	Proposed effects	Reference(s)
SOD1 (↑)	ROS elimination	[24, 28]
SOD2 (↑)	ROS elimination	[28]
GPx (↑)	ROS elimination	[25]
Catalase (↑)	ROS elimination	[25]
iNOS (↓)	Decreases in reactive radicals	[25, 69, 70]
nNOS (↓)	Decreases in reactive radicals	[25]
GST (↑)	Elimination of ROS-derived reactive metabolites	[30]
NQO1 (↑)	Elimination of ROS-derived reactive metabolites	[30]
Seladin-1 (↑)	Antiapoptosis	[33, 34]
Neuroglobin (↑)	Antiapoptosis, anti-inflammation	[40, 41]
IL-6 (↓)	Anti-inflammation	[41]
IP-10 (↓)	Anti-inflammation	[41]
MMP-9 (↓)	Anti-inflammation	[25, 69, 70]
Cytochrome *c* oxidase (↑)	Increment of mitochondrial efficiency	[50]
Bax (↓)	Antiapoptosis	[42]

IL-6: interleukin-6; IP-10: IFN-gamma-inducible protein 10; MMP-9: matrix metalloproteinase-9; NOS: nitric oxide synthase; NQO1, NADPH: quinone oxidoreductase 1.

## Abbreviations

CNS: Central nervous system
ER: Estrogen receptor
ERK: Extracellular signal-regulated kinase
GABA: Gamma-aminobutyric acid
GPx: Glutathione peroxidase
GST: Glutathione S-transferase
MeHg: Methylmercury
PR: Progesterone receptor
ROS: Reactive oxygen species
SOD: Superoxide dismutase
TBT: Tributyltin.
